# Innate Immune Response to Fasting and Refeeding in the Zebrafish Kidney

**DOI:** 10.3390/biom11060825

**Published:** 2021-06-01

**Authors:** Zongzhen Liao, Dihang Lin, Jirong Jia, Ran Cai, Yang Yu, Wensheng Li

**Affiliations:** State Key Laboratory of Biocontrol, Guangdong Province Key Laboratory for Aquatic Economic Animals, Guangdong Provincial Engineering Technology Research Center for Healthy Breeding of Important Economic Fish, School of Life Sciences, Sun Yat-Sen University, Guangzhou 510275, China; liaozzh@mail2.sysu.edu.cn (Z.L.); lindihang2018@sibcb.ac.cn (D.L.); jiajr3@mail.sysu.edu.cn (J.J.); Alan6382@163.com (R.C.); yuyang65@mail2.sysu.edu.cn (Y.Y.)

**Keywords:** innate immune system, kidney, oxidative stress, compensatory growth, zebrafish

## Abstract

Animals acquire nutrients and energy through feeding to achieve a balance between growth and organismal health. When there is a change in nutrient acquisition, the state of growth changes and may also cause changes in the intrinsic immune system. Compensatory growth (CG), a specific growth phenomenon, involves the question of whether changes in growth can be accompanied by changes in innate immunity. The zebrafish (*Danio rerio*), a well-known fish model organism, can serve as a suitable model. In this study, the zebrafish underwent 3 weeks of fasting and refeeding for 3 to 7 day periods. It was found that CG could be achieved in zebrafish. Zebrafish susceptibility to *Streptococcus agalactiae* increased after starvation. In addition, the amount of melano-macrophage centers increased after fasting and the proportion of injured tubules increased after refeeding for 3 and 5 days, respectively. Furthermore, the kidneys of zebrafish suffering from starvation were under oxidative stress, and the activity of several antioxidant enzymes increased after starvation, including catalase, glutathione peroxidases and superoxide dismutase. Innate immune parameters were influenced by starvation. Additionally, the activity of alkaline phosphatase and lysozyme increased after starvation. The mRNA expression of immune-related genes like *il-1β* was elevated to a different extent after fasting with or without lipopolysaccharides (LPS) challenge. This study showed that the function of the innate immune system in zebrafish could be influenced by nutrition status.

## 1. Introduction

Basic life activities are dependent on the energy supply of the organism. The nutrient state of the animal body can influence the functioning of the immune system [1,2]. Compensatory growth (CG) is a period of rapid growth after a phase of growth suppression, which can be caused by a shortage of food and other environmental factors [3,4]. This phenomenon was first recorded in mammals and was eventually found in birds [3] and teleosts [5]. CG is divided into two phases: the catabolic phase and the hyper anabolic phase [6]. One of the basic patterns of CG is fasting and refeeding. After refeeding, fish can experience partial, full, over- or no compensation [4]. The special feeding regime of CG is regarded as a potential way to improve production efficiency in farmed species [7].

The innate immune response is the first boundary of host defense, protecting organisms from pathogens. In fish, organs of the immune system include the thymus, head kidney, caudal kidney, gills, liver and gut [8,9]. A broad range of cell types take part in nonspecific cellular defense responses in fish, including monocytes/macrophages, nonspecific cytotoxic cells and granulocytes (neutrophils). Immunometabolism is the interplay between immunological and metabolic processes [10]. The metabolic status of fish can affect their immune function. In humans, the nutritional state influences immune cell counts—and their activity [11]. It was found that the function of the immune system in mice could be affected by their metabolic state [12,13,14]. Intermittent fasting in crucian carp (*Carassius auratus*) changed the gut bacterial communities and increased the activity of super oxide dismutase (SOD) and lysozyme [15]. Starvation also alters the gene expression of innate immune responses in the livers of salmon [16]. In addition, studies on the effects of the metabolic state on the immune system can be found in other teleosts [17,18,19]. However, the influence of the metabolic state upon the immune system in teleosts is still poorly understood. Oxidative stress can limit the function of the innate immune system and induce components like toll-like receptors [20,21]. Meanwhile, starvation can increase the production of ROS and cause autophagy [22,23]. It would be worth exploring whether oxidative stress mediates the relationship between starvation and immunity.

Zebrafish (*Danio rerio*) area widely applied model in the study of immunity and disease. The innate immune systems of zebrafish begin to develop on day 1 of embryogenesis, and the adaptive immune system is absent until 4–6 weeks after fertilization. This makes zebrafish a great model for studies of the innate immune system [9]. The kidney is the primary lymphoid organ in zebrafish [24]. The *havcr1* gene encodes a transmembrane tubular protein called kidney injury molecule-1 (KIM-1) [25]. KIM-1 is the indicator of tissue damage in the kidney, and the overexpression of KIM-1 causes kidney injury in zebrafish [26]. It was found that fasting could change the metabolic state in zebrafish [27]. Fasting led to decreased oxygen consumption and liver lipid and glycogen contents. It also increased the activity of β-oxidation and gluconeogenesis in liver. Meanwhile, reactive oxygen species (ROS) took part in the CG in zebrafish [28]. ROS, produced in the liver, were necessary for CG in zebrafish because they induced the AMPK pathway. In the present study, we adopted a strategy of fasting and refeeding to explore the pattern of CG in zebrafish. *Streptococcus agalactiae* caused severe sepsis and meningitis in zebrafish and could infect more than 30 species of fish [29]. We chose *Streptococcus agalactiae* and lipopolysaccharides (LPS) for injection to explore the influence of CG on the innate immune system, combined with changes in immune-related parameters and gene expression.

## 2. Materials and Methods

### 2.1. Zebrafish, Feeding Regimes and Body Weight

The adult zebrafish used in the experiment were purchased from Yuehe Fish-Bird-Flower market (Guangzhou, China) and acclimatized for two weeks. All fish rearing was performed at the fish culture experiment station at Sun Yat-sen University, Guangdong Province, China. All animal experiments were performed in accordance with the guidelines and approval of the Sun Yat-Sen University Animal Care and Use Committee. During the experimental period, pH ranged from 7.8 to 7.9, dissolved oxygen ranged from 6.95 to 7.23 mg/L, and water temperature ranged from 26.5 to 28 °C. Concentrations of ammonia-N and nitrite nitrogen were maintained lower than 0.2 and 0.005 mg/L, respectively. Salinity of water was 0.2 ppt. Zebrafish were maintained in a 12:12-h light-dark cycle. To explore the changes in body weight during fasting and refeeding, body weight was recorded once a week. The initial body weight of the zebrafish in the experiment were 0.35 ± 0.06 g, and they were aged approximately 3 months. The zebrafish were separated randomly into 24 L tanks, and each tank contained 30 fishes. They were offered commercial diet twice daily with satiation feeding. Feeding regimes for each experiment are shown in Table 1. After treatment, zebrafish were anesthetized by overdose MS-222 (Sigma, Burlington, MA, USA) and sacrificed. Then, the kidney was removed to Eppendorf tube and placed into liquid nitrogen immediately. The sample was transferred from liquid nitrogen and stored at −80 °C.

### 2.2. Streptococcus agalactiae Challenge

The zebrafish undergoing fasting and refeeding were challenged with *Streptococcus* *agalactiae*. The *Streptococcus agalactiae* strain was kindly provided by Professor Anxing Li, School of Life Sciences, Sun Yat-sen University [30]. The zebrafish in each group were further separated into four small groups, with 15 fish in each small group. The zebrafish in three of these groups were anesthetized by 80 mg/L MS-222 and intraperitoneally injected with 5 μL containing 5 × 10^6^ colony-forming units of *Streptococcus agalactiae*. The remaining zebrafish were injected with 5 μL of PBS. All of the zebrafish were observed for 4 days without feeding. The mortality rate was recorded.

### 2.3. LPS Injection

The zebrafish undergoing fasting and refeeding (F24, S24 and F21R3) were challenged with LPS (Sigma, Burlington, MA, USA). LPS powder was dissolved in sterile water to a final concentration of 1 mg/mL. Before use, 1 mg/mL LPS was diluted with PBS, and the final concentration was 20 μg/mL. The zebrafish in each group were separated into two small groups, and there were 12 fish in each small group. The fish in the two groups were injected with LPS and PBS. The zebrafish were anesthetized with MS-222 and intraperitoneally injected with 5 μL of 20 μg/mL LPS or PBS. After 3 h, zebrafish were anesthetized and sacrificed. After that, the kidney was removed and kept at −80 °C.

### 2.4. Histopathology

The zebrafish were maintained with refeeding for 14 days after starvation for 21 days. Samples were taken at days 21, 24, 28 and 35. After sacrifice, zebrafish kidneys were removed and immersed in 4% formaldehyde. Then, the kidneys underwent paraffin sectioning. The sections were stained with hematoxylin eosin (HE) (Beyotime, Shanghai, China) and observed with a light microscope (Olympus, Tokyo, Japan). To quantify the change of injured tubules and melano-macrophage centers (MMCs), 5 non-overlap pictures were captured from each slide. The areas of injured tubules and the whole sample were measured using Cellsens software (Olympus, Tokyo, Japan). From each picture, the proportion of injured tubules was calculated by dividing the area of injured tubules by the area of the whole sample. The quantity of MMCs was counted in each picture, then divided by the area of the whole sample. Then, the fold changes of the MMCs were calculated with group F21 as the standard.

### 2.5. Related Biochemical Parameter Assay

The contents of malondialdehyde (MDA) and nitric oxide (NO) and the enzymatic activities of SOD, alkaline phosphatase (ALP), catalase (CAT), glutathione peroxidases (GSH-Px) and lysozyme in the kidney were measured with corresponding commercial kits in accordance with the manufacturer’s protocols. In brief, kidneys removed from zebrafish were placed into liquid nitrogen and stored at −80 °C. Before testing, each kidney was taken out and made into homogenate in pre-cold PBS with tissue disruptor. Kidney homogenate was centrifuged at 4 °C and 12,000× *g* for 10 min and the supernatant was taken as kidney extract for detection. The MDA content was detected with a Lipid Peroxidation MDA Assay Kit (Beyotime, Shanghai, China) and the detection was based on the color reaction between MDA and thiobarbituric acid. The wavelength used was 535 nm. The NO content was measured by a Griess reagent system (Promega, Madison, WI, USA). The Griess reagent system is based on the nitrite reaction with sulfanilamide and N-1-napthylethylenediamine dihydrochloride under acidic condition. The NO content was calculated through a sodium nitrite standard and normalized to protein content. Absorbance was measured with a filter at 490 nm. The activity of lysozyme was detected with a Lysozyme Assay Kit (Thermo Fisher, Waltham, MA, USA) following the manufacturer’s protocol. Kidney extract was incubated with fluorescein-conjugated *Micrococcus lysodeikticus* cell wall. Fluorescence was measured after 45 min of incubation at 37 °C, at excitation and emission wavelengths of 485 and 535 nm. The value of lysozyme was calculated through a standard curve and normalized to protein content. The enzymatic activities of SOD, ALP, CAT and GSH-Px were separately detected with SOD typed assay kits, alkaline phosphatase assay kits, catalase assay kits and glutathione peroxidase assay kits (Nanjing Jiancheng, Nanjing, China). A unit of SOD activity was defined as the amount of SOD corresponding to 50% inhibition of the nitrobule tetrazolium reduction rate per mg of protein in 1 mL system. The wavelength used was 570 nm. A unit of ALP activity was defined as the volume of enzyme that reacted with the substrate to produce 1 mg of phenol in 30 min at 37 °C and normalized to protein content. The wavelength used was 490 nm. The CAT activity was based on the consumption of hydrogen peroxide. The wavelength used was 405 nm. The activity of GSH-Px was measured by the rate of catalyzing reaction between hydrogen peroxide and GSH. Subtracting the non-enzymatic reaction, a unit of GSH-Px activity was defined as the reduction of 1 mol/L in GSH concentration in each system. The wavelength used was 405 nm. The concentration of protein was measured with a BCA Protein Assay Kit (Beyotime, Shanghai, China). The wavelength used was 570 nm.

### 2.6. ROS Production

The content of ROS in the kidney was determined with the fluorescent probe 2′,7′-dichlorofluorescein diacetate (DCF-DA) (Sigma, Burlington, MA, USA) as previously described [28]. In brief, kidney removed from zebrafish was placed into pre-cold 100 μL PBS and made into homogenate with tissue disruptor. After centrifugation for 10 min with 10,000× *g* in 4 °C, 20 μL of kidney extract was added to 96-well black microplates, and 25 μM of DCF-DA was added to each well. The microplate was incubated at 37 °C for 30 min. Fluorescence was measured in a multilabel plate reader (PerKinElmer Victor X5, Waltham, MA, USA) with excitation and emission filters set at 485 and 535 nm. The protein content of kidney extract was measured with a BCA Protein Assay Kit. The content of ROS for each sample was calculated by normalizing the fluorescence value to the protein content. The concentration of protein was measured with a BCA Protein Assay Kit (Beyotime, Shanghai, China). The wavelength used was 570 nm.

### 2.7. Respiratory Burst Assay

Zebrafish undergoing fasting and refeeding (F24, S24 and F21R3) were used to measure respiratory burst activity. The protocol for the respiratory burst assay was performed according to a previously published method [31,32]. In brief, the kidneys were separated from the anesthetized zebrafish and placed in 96-well microplates with 100 μL of Dulbecco’s modified Eagle’s medium (DMEM)/F-12. After incubation of the plate for 30 min at 28 °C, 100 μL of DMEM/F-12 containing DCF-DA, dimethylsulfoxide (DMSO) and phorbol myristate acetate (PMA) (Thermo Fisher, Waltham, MA, USA) was added to each detected well. Meanwhile, 100 μL of DMEM/F-12 containing DMSO was added to each control well. The final concentrations of the different solutions were as follows: 500 ng/mL DCF-DA, 0.2% DMSO and 200 ng/mL PMA. Fluorescence was measured in a multilabel plate reader with excitation and emission filters set at 485 and 535 nm. The fluorescence of each well was measured immediately after the addition of solutions to the well and every 15 min thereafter for 150 min. The time point to compare fluorescence values was 150 min. The fluorescence values of the uninduced control group were subtracted from the fluorescence values of the individual PMA-induced groups. The normalized fluorescence values were calculated. These normalized fluorescence values were sorted into three columns.

### 2.8. RNA Extraction and Real-Time PCR

The frozen kidneys were ground in TRIzol (Omega, Norcross, GA, USA) and total RNA was separated according to protocol [33]. After digestion with DNase1 (NEB, Ipswich, MA, USA), cDNA was produced by a RevertAid First Strand cDNA Synthesis Kit (Thermo Fisher, Waltham, MA, USA) with Random Hexamer Primer. qRT-PCR was set up in 10 μL reaction mixtures containing 5 μL of 2× SYBR Green qPCR Master Mix (Bimake, Houston, TX, USA). All samples were run in duplicate and normalized to *eEF1a1a* expression. The sequences of the oligonucleotide primers used in the present study are described in Supplemental Table S1. The relative mRNA quantification of the target to the reference was determined using the 2^−ΔΔCT^method [34].

### 2.9. Statistical Analysis

Quantitative data are shown as the mean value ± SD. Statistical analyses were performed with SPSS version 24 (SPSS Inc., Chicago, IL, USA). The statistical comparisons between two groups were carried out using Student’s t-test or nonparametric test, depending on the result of the test of normality. The analyses for multiple groups with normal distribution were performed using one-way ANOVA, and the rest were performed using nonparametric tests. All the statistics data and methods can be checked in Table S2.

## 3. Results

### 3.1. Changes in Body Weight and Antibacterial Properties of Zebrafish during Fasting and Refeeding

**Figure 1:**
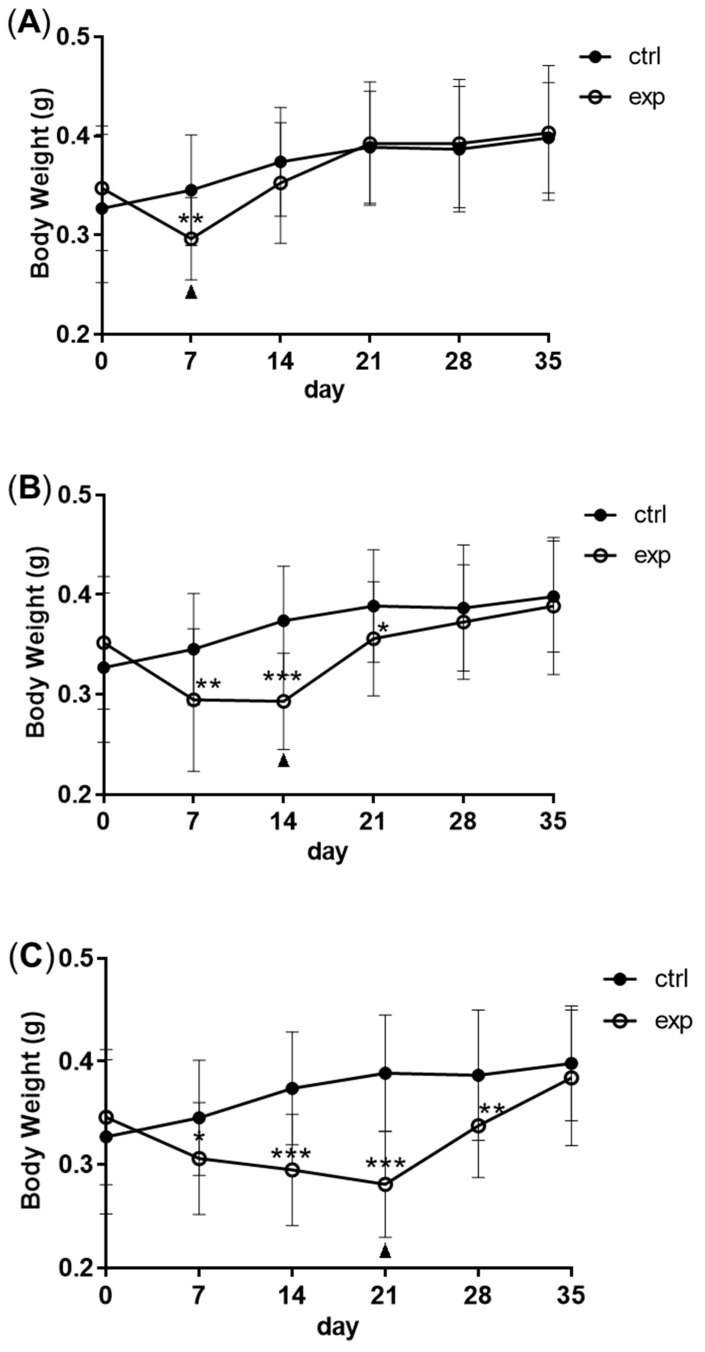

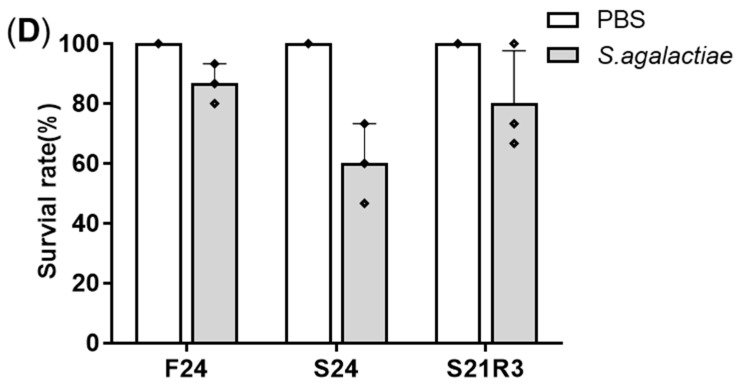
The model of compensatory growth in zebrafish. A to C: the body weight change of zebrafish during fasting and refeeding. (**A**) The zebrafish were fasted for 7 days and refed for 28 days. (**B**) The zebrafish were fasted for 14 days and refed for 21 days. (**C**) The zebrafish were fasted for 21 days and refed for 14 days. *n* = 21–22. (**D**) The survival rate of zebrafish which were intraperitoneally injected with *S. agalactiae*; F: feeding; S: starvation; R: refeeding. *n* = 3, *: *p* < 0.05, **: *p* < 0.01; ***: *p* < 0.001.

Full compensatory growth was realized in zebrafish. After fasting for 7 to 14 days, zebrafish lost at least 15% body weight. Following 14 days of refeeding, the body weight of zebrafish in the fasting group caught up with that of the control group (Figure 1A–C). After fasting and refeeding, in order to explore the resistance of zebrafish to pathogens, they were intraperitoneally injected with *Streptococcus agalactiae*. Zebrafish in the fasting group had an increased mortality rate compared with the control group. With the extension of refeeding, the mortality rate continued to decrease (Figure 1D).

### 3.2. Morphologic Alterations of the Kidneys of Zebrafish during Fasting and Refeeding

**Figure 2:**
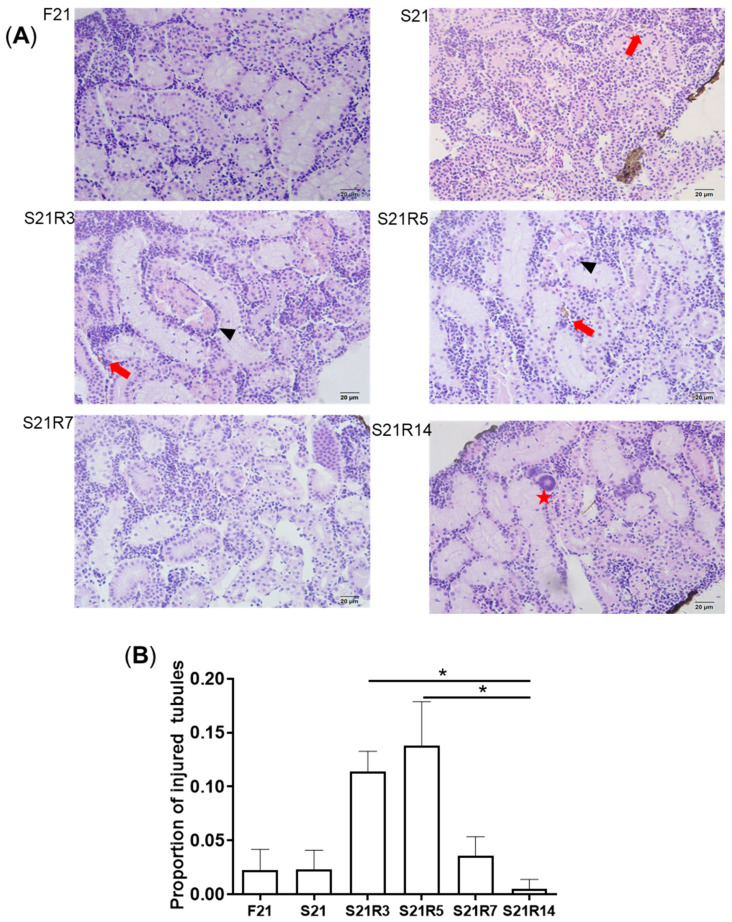

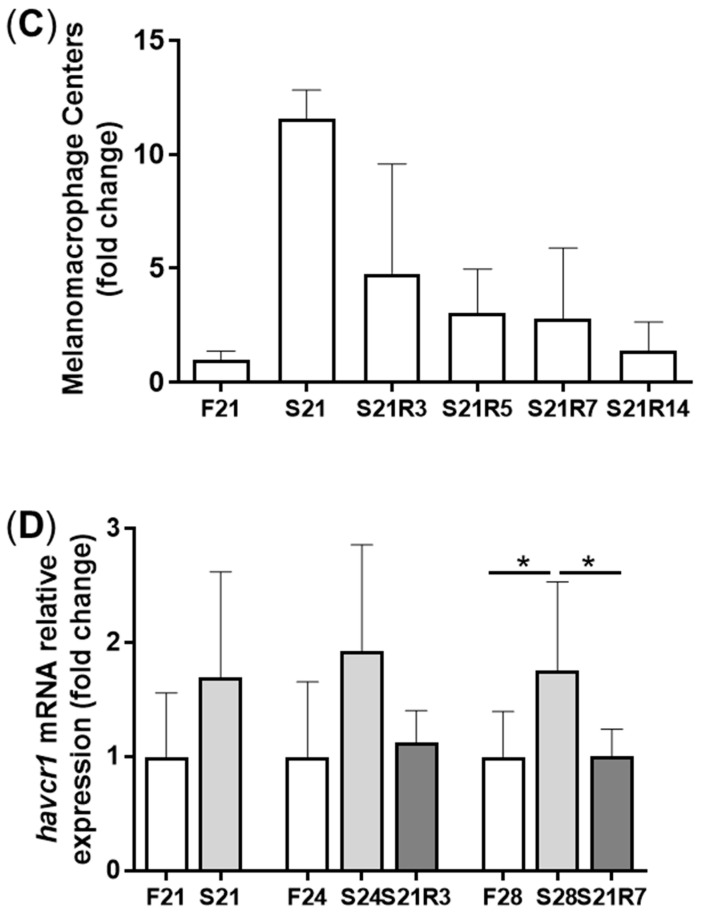
Morphological changes in the kidneys of zebrafish during fasting and refeeding. (**A**) HE-staining of kidney in zebrafish during fasting and refeeding. Black triangle denotes injured tubules. Red arrow denotes MMCs. Red star denotes hyaline droplets. (**B**) Statistic analysis of proportion of injured tubules in kidney. (**C**) Statistic analysis of MMCs in kidney. *n* = 3–4. (**D**) The mRNA relative expression of *havcr1* gene in kidney of zebrafish during fasting and refeeding. *n* = 8–10. F: feeding; S: starvation; R: refeeding. *: *p* < 0.05.

Kidney is the main immune tissue in zebrafish. Starvation caused morphological changes in the kidneys. After 21 days of fasting, the kidney exhibited obvious morphological changes. We found that more injured tubules with hyaline droplets appeared after refeeding for 3 and 5 days. This situation improved at 7 days. In addition, it was observed that the number of melano-macrophage centers (MMCs) in the kidney increased after starvation (Figure 2A–C). Along with refeeding, this symptom was rescued. The mRNA expression of havc*r1* increased after fasting (Figure 2D), showing that starvation may cause kidney injury.

### 3.3. Oxidative Stress Was Triggered during Fasting and Refeeding

**Figure 3:**
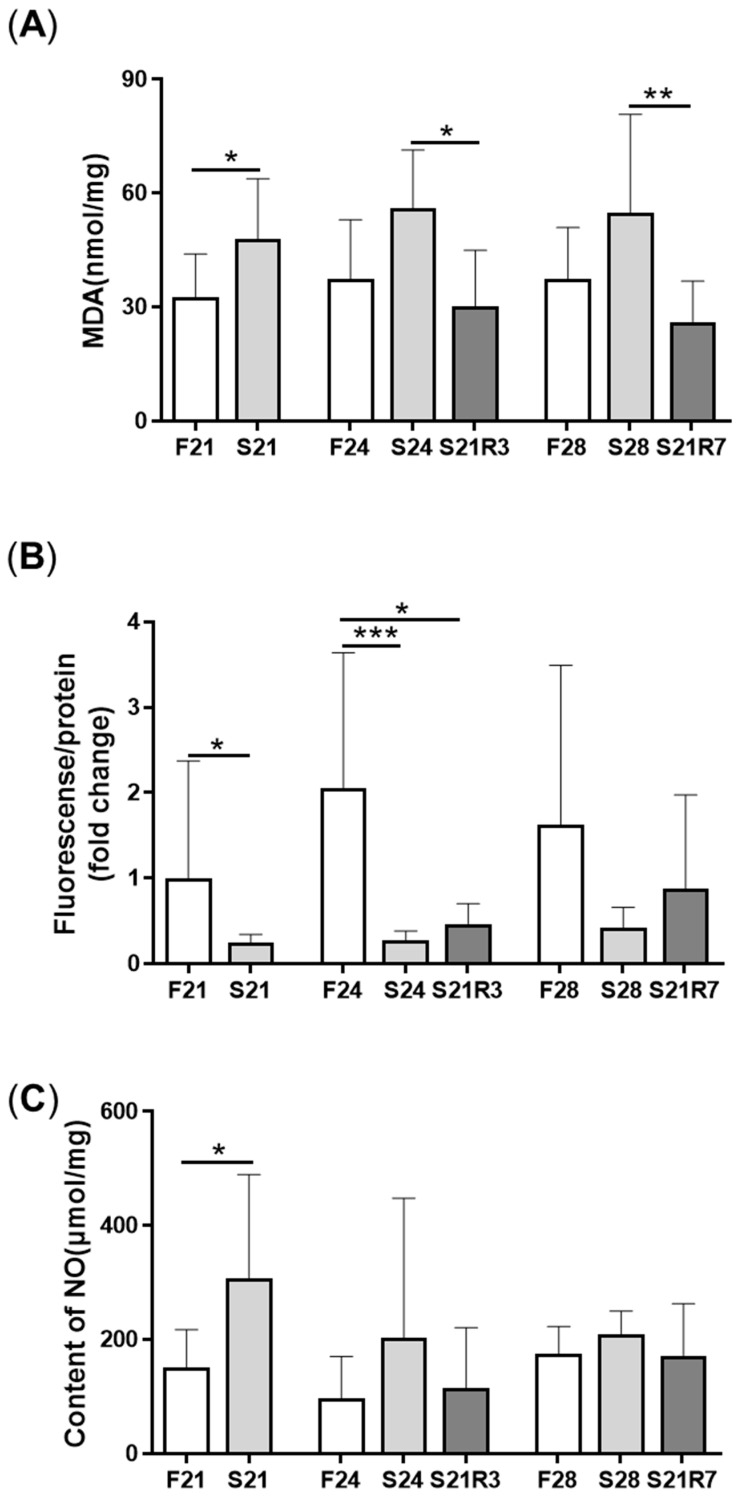
Oxidative stress in the kidneys of zebrafish during fasting and refeeding. (**A**) The content of MDA in kidney. (**B**) The change of ROS in kidney. (**C**) The content of NO in kidney. F: feeding; S: starvation; R: refeeding. *n* = 6–10. *: *p* < 0.05, **: *p* < 0.01; ***: *p* < 0.001.

**Figure 4:**
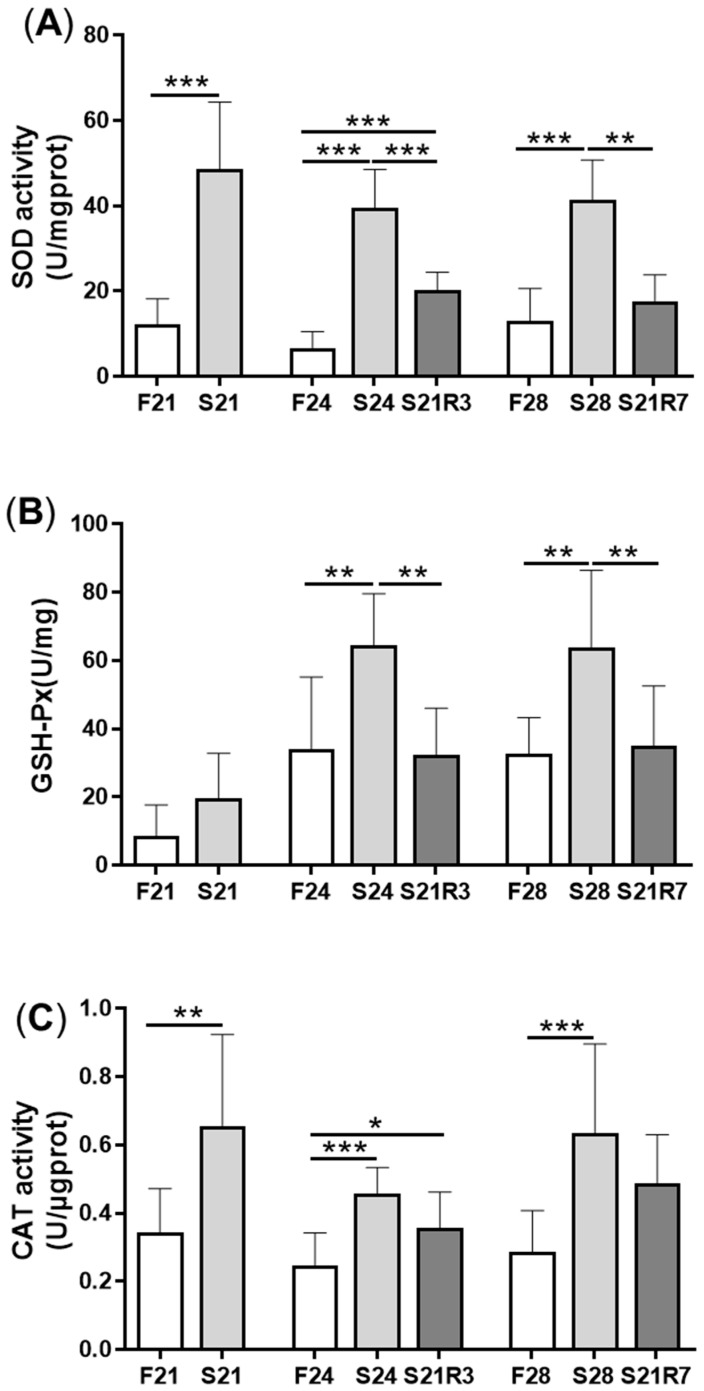
The activity of enzymes in the antioxidant systems in kidneys of zebrafish during fasting and refeeding. (**A**) The activity of SOD in kidney. (**B**) The activity of GSH-Px in kidney. (**C**) The activity of CAT in kidney. F: feeding; S: starvation; R: refeeding. *n* = 6–10. *: *p* < 0.05, **: *p* < 0.01; ***: *p* < 0.001.

MDA content was enhanced significantly after fasting for 21 and 24 days and decreased after 3 days of refeeding (Figure 3A). However, the content of ROS decreased with 24 days of starvation and recovered with refeeding for 7 days (Figure 3B). The content of NO increased after fasting for 21 days (Figure 3C). The activity of antioxidant enzymes, including SOD, GSH-Px and CAT, increased after fasting. The activities of GSH-Px and CAT rapidly decreased and recovered at day 3 of refeeding. Although the activity of SOD declined with refeeding, it was still higher than that of the control (Figure 4).

### 3.4. The Innate Immune System of the Zebrafish Kidney Was Affected by Fasting and Refeeding

**Figure 5:**
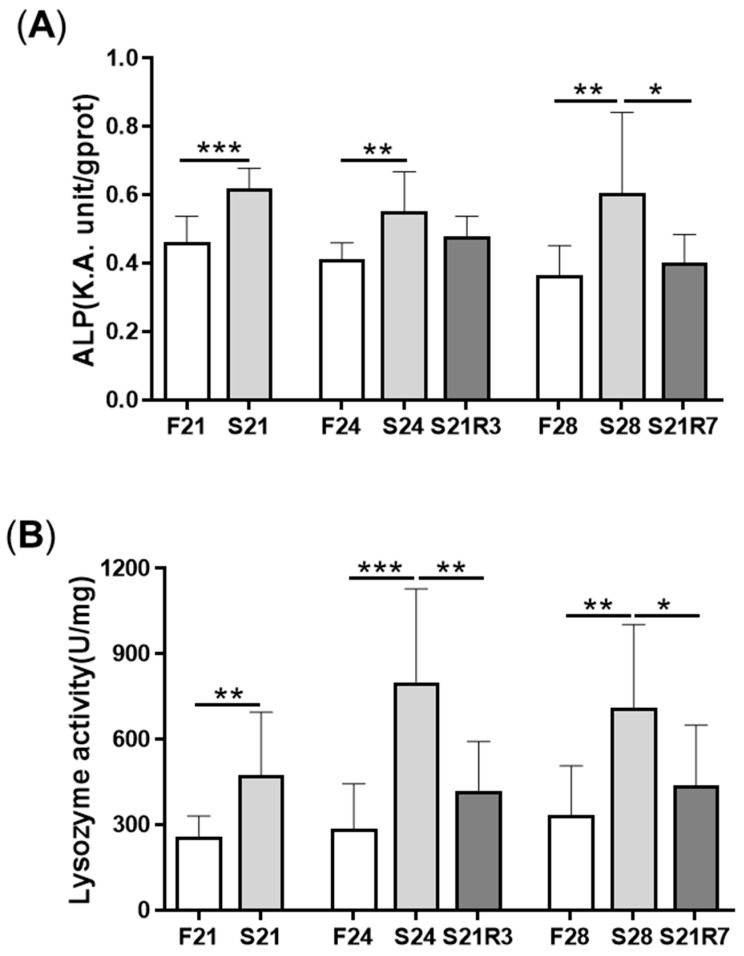

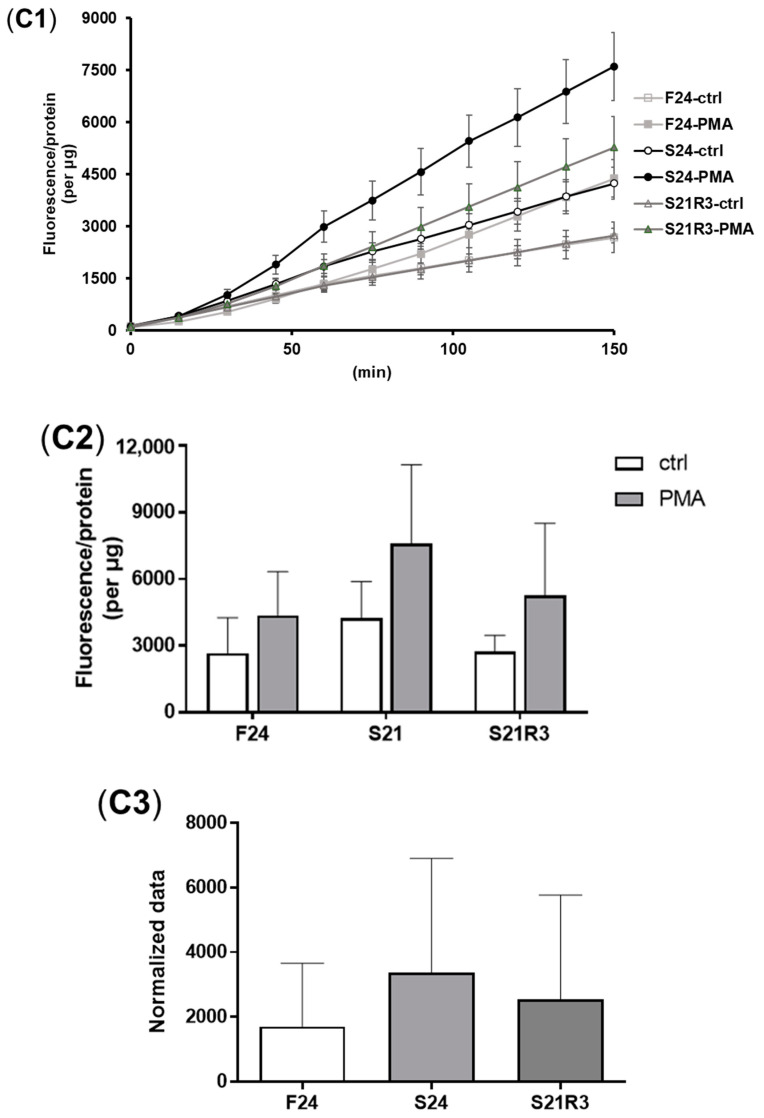
The parameters of the innate immune system in the kidneys of zebrafish during fasting and refeeding. (**A**) ALP activity in kidney of zebrafish during fasting and refeeding. (**B**) Lysozyme activity in kidney of zebrafish during fasting and refeeding. (**C**) The respiratory burst response of kidney in zebrafish after fasted and refed. (**C1**) The value of fluorescence/protein of kidney at different times. (**C2**) Fluorescence/protein values of kidney at 150 min. (**C3**) Normalization of the value of fluorescence/protein of kidney at 150 min. F: feeding; S: starvation; R: refeeding. *n* = 12. *: *p* < 0.05, **: *p* < 0.01; ***: *p* < 0.001.

**Figure 6:**
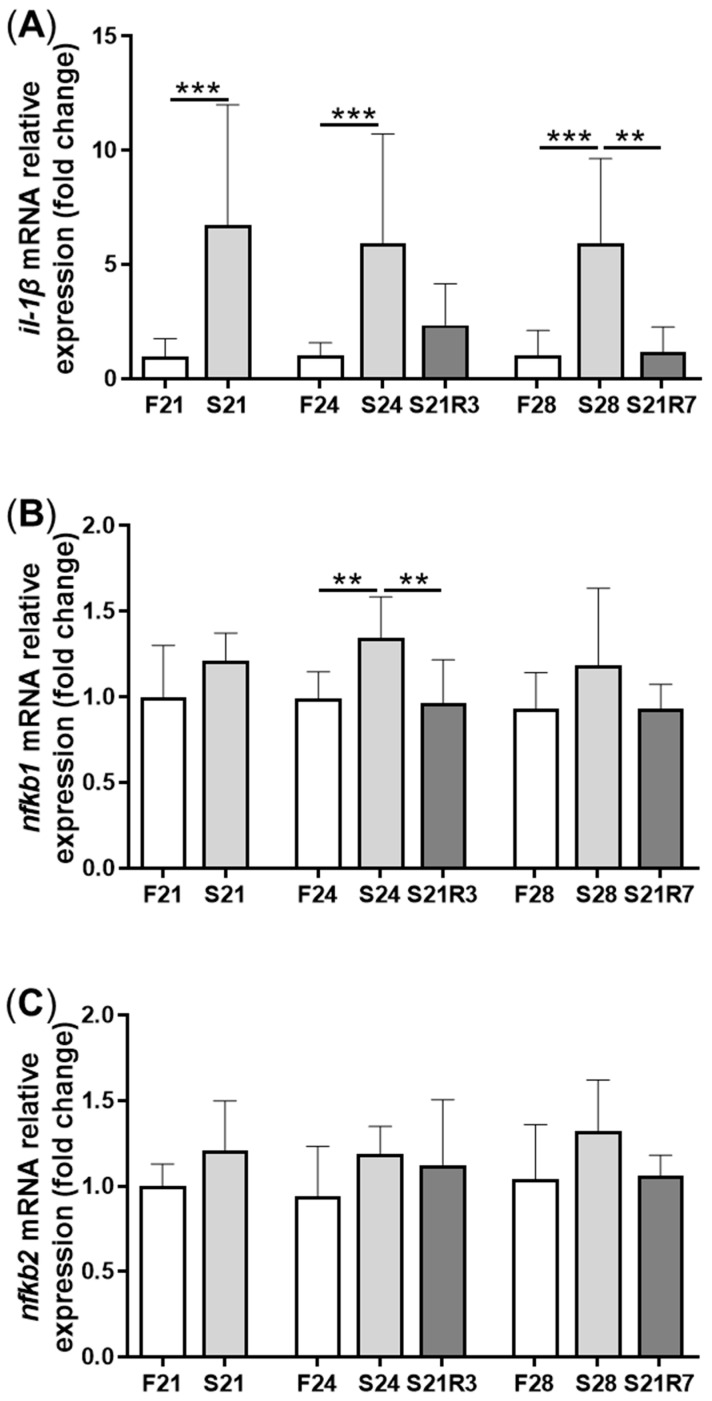

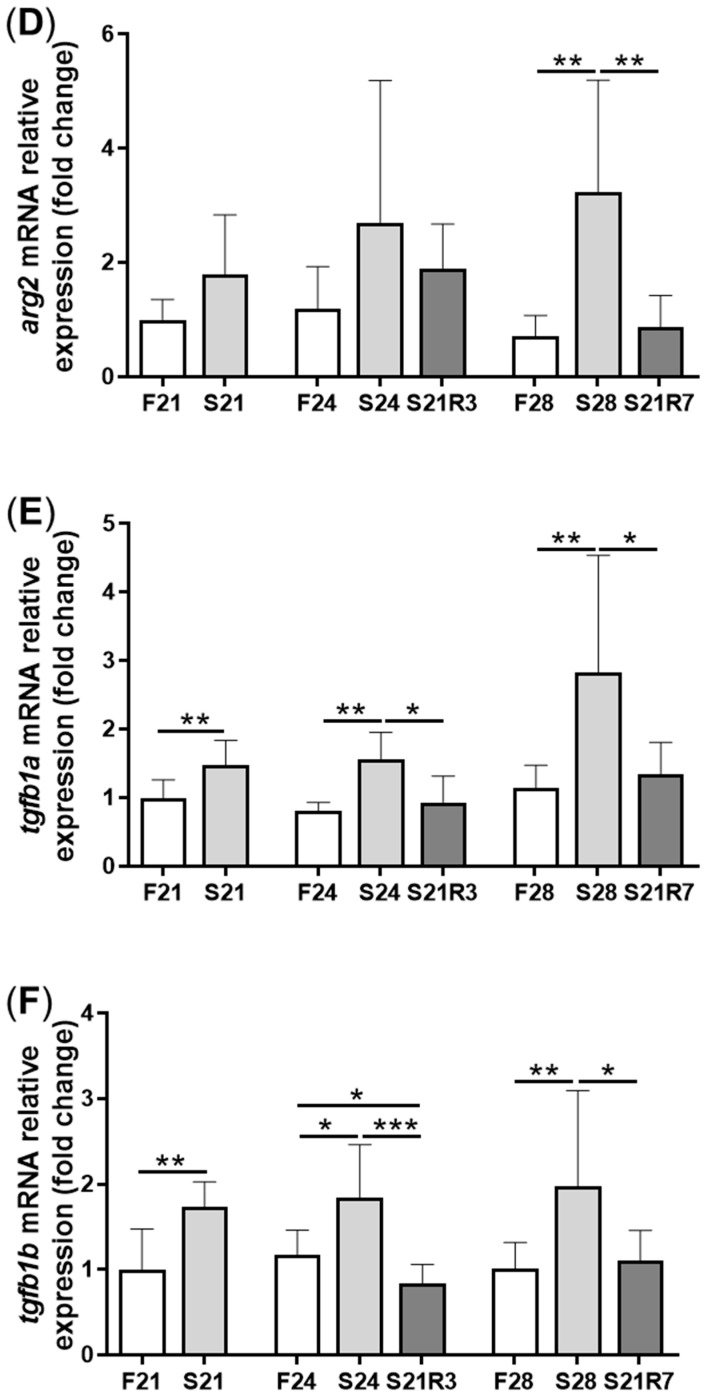
The mRNA relative expression of immune-related genes in the kidneys of zebrafish during fasting and refeeding. (**A**–**F**):*il-1β*, *nfκb1*, *nfκb2*, *arg2*, *tgfb1a* and *tgfb1b*. F: feeding; S: starvation; R: refeeding. *n* = 8–10. *: *p* < 0.05, **: *p* < 0.01; ***: *p* < 0.001.

**Figure 7:**
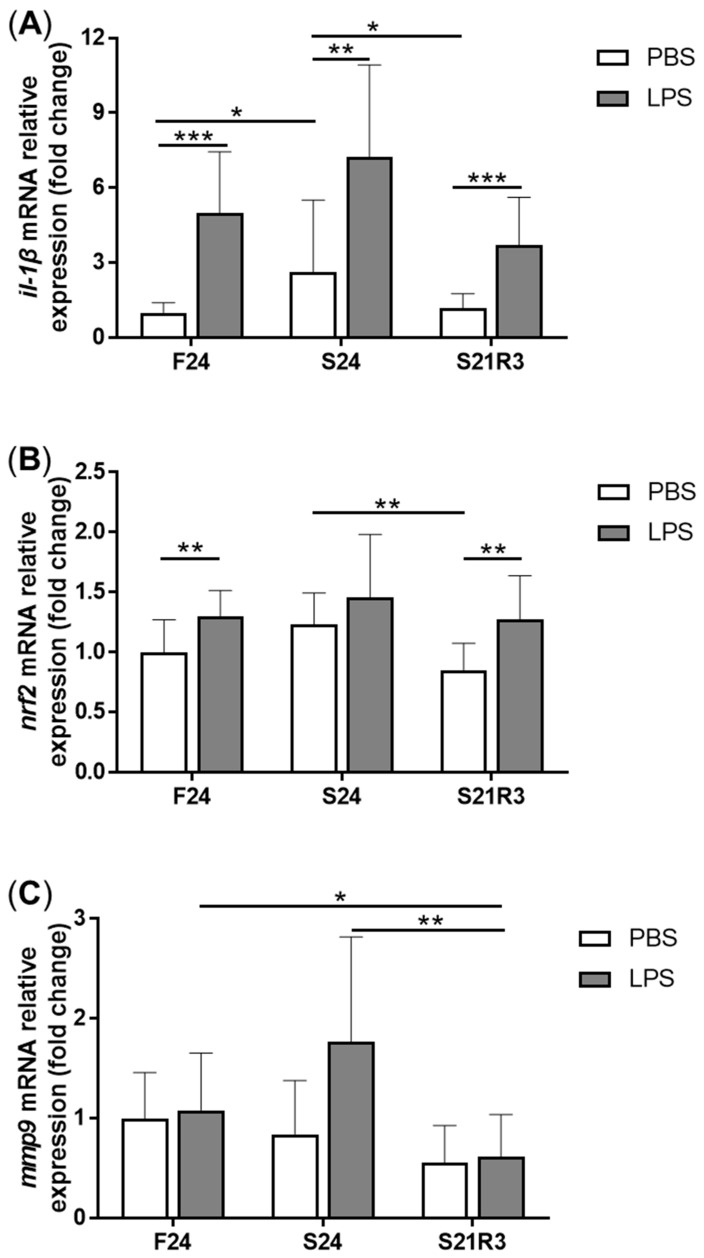
The mRNA relative expression of immune-related genes in the kidneys of zebrafish challenged with 5 μL of 20 μg/mL LPS after fasting and refeeding. (**A**–**C**) *il-1β*, *mmp9* and *nfκb1*. F: feeding; S: starvation; R: refeeding. *n* = 8–12. *: *p* < 0.05, **: *p* < 0.01; ***: *p* < 0.001.

The activity of ALP increased in the kidney after fasting and decreased after refeeding (Figure 5A). With refeeding for 7 days, there was no significant difference between the refeeding group and the control. The activity of lysozyme in the kidney showed a similar trend to ALP, which increased with fasting (Figure 5B). It declined rapidly after refeeding. The respiratory burst response in the kidney showed no significant changes in different groups, but the response was higher in the fasting and refeeding group (Figure 5C). The mRNA expression of the proinflammatory cytokine *il-1β* showed a remarkable increase in all fasting groups but promptly declined after refeeding (Figure 6A). The mRNA expression of *tgfb1a* and *tgfb1b* showed the same trend as *il-1β* (Figure 6E,F). However, the mRNA expression of *nfκb1* only notably increased after starvation for 24 days and decreased after refeeding for 3 days. The mRNA expression of *nfκb2* showed a trend similar to that of *nfκb1* (Figure 6B,C). The gene *arg2* encodes arginase Ⅱ, which is related to the proinflammatory response. In addition, the mRNA expression of *arg2* increased remarkably in all fasting groups and was reduced after refeeding (Figure 6D). After challenging the zebrafish who went through fasting and refeeding, the response was stronger in the fasting group. The mRNA expression of *il-1β* and *mmp9* in LPS-treated fasting group was significantly higher than that in the LPS-treated control or refeeding groups (Figure 7).

## 4. Discussion

In the present study, we found that full CG could be realized in zebrafish after different durations of starvation. Even after 3 weeks of fasting, the body weight of zebrafish reached that of the control group after two weeks. This result was consistent with previous work [28]. Studies have shown that starvation and refeeding influence the metabolic state of zebrafish [27,28]. In farmed species, the significance of CG is that it improves the efficiency of production. It is important for farmed species to maintain antibacterial properties during fasting and refeeding. During this process, zebrafish have the potential to suffer from pathogens. It was reported that starvation did not influence the immune response in infected *Piaractus mesopotamicus* [18]. Unfortunately, fasting promoted zebrafish susceptibility to *S. agalactiae*. Simultaneously, biochemical parameters and mRNA expression of the innate immune system were influenced by starvation.

In zebrafish, lymph nodes are absent, and the kidney is the primary lymphoid organ, equivalent to mammalian bone marrow [24]. In adult zebrafish, the kidney is the major site of hematopoiesis [35]. The populations in kidney marrow contain many hematopoietic cell types, including macrophages, neutrophils and lymphocytes [36,37]. The proper function of the kidney is important for the innate immune system in zebrafish. In the present study, we observed some morphological changes in the kidneys of zebrafish subjected to starvation. The deposition of MMCs in the kidney significantly increased after starvation. It was previously found that starvation could cause the deposition of melano-macrophages (MMs) in the kidney and spleen in fish [38]. MMCs mainly appear in the kidney, liver and spleen and are diffuse and less structured in the kidney [39]. MMCs are considered to function in both immune defenses and normal, nonimmunological, physiological processes. The primary function of MMCs is phagocytosis. It is thought that MMCs may take part in both innate and adaptive immune responses. The addition of MMCs after starvation may be due to hemophagocytosis and clearance of cell debris. In addition, in the early days of refeeding, the structure of hyaline droplets was observed. The presence of hyaline droplets is thought to be indicative mainly of alpha_2u_-globulin in tubular epithelial cells [40]. In addition, in a model of histiocytic sarcoma, neoplastic histiocytes produced lysozyme, and lysozyme accumulated in tubular epithelial cells [41]. In summary, the formation of hyaline droplets occurs because the kidney cannot digest the protein that accumulates in tubular epithelial cells. The level of KIM-1 (encoded by *havcr1*) can indicate the structural damage of tubules [42]. After starvation, the mRNA expression of *havcr1* notably increased and decreased with refeeding. We can predict that the function of the kidney is impaired by fasting, and with refeeding, kidney function recovers. However, the requirements are burdensome at the beginning of refeeding.

Phagocytosis is an important process of defense in the innate immune system in fish. Oxidative stress will limit the immune response [20]. Antioxidant defense mainly depends on enzymes. SOD, GPx and catalase are important components of antioxidant systems [43]. SOD mediates the conversion of superoxide anion and H_2_O_2_. Catalase takes part in the detoxification of H_2_O_2_ to water. GPx catalyzes hydroperoxides (e.g., H_2_O_2_ and lipid hydroperoxides) to H_2_O by oxidizing glutathione (GSH) into glutathione disulfide (GSSH) [43,44,45]. MDA is one of the main lipid oxidation products and is produced from polyunsaturated fatty acids (PUFAs) by chemical reactions and reactions catalyzed by enzymes. ROS can peroxidize PUFAs to generate MDA. MDA content is a biomarker commonly used to assess oxidative stress [46]. In the present work, even though the content of ROS decreased, the content of MDA in the kidney increased remarkably, and the activity of all enzymes belonging to the antioxidant system was also elevated. The detection of ROS is a transient response. The change of activity of antioxidant enzyme was the result of long-term fasting. The contrary trends between ROS and antioxidant enzymes may be due to the fact that normal cellular activity processes produce ROS, while prolonged starvation leads to diminished cellular activity. This result showed that starvation caused oxidative stress in the kidney in a chronic manner. Persisting oxidative stress may cause damage to substances in cells such as lipids and proteins and finally lead to apoptosis and the accumulation of oxidized molecular aggregates [20]. In teleosts, it was reported that starvation induced the content of MDA in the liver and intestine and increased the activity of antioxidant enzymes [47,48,49]. These results implied that although the antioxidant system was activated, it was not sufficient to clear the damage caused by starvation. Meanwhile, oxidative stress can activate a large range of transcription factors, such as NF-κB, Nrf2 and HIF-1α. Subsequently, these transcription factors induce the expression of many cytokines and chemokines. Thus, oxidative stress can lead to chronic inflammation [44].

Challenged with *Streptococcus agalactiae*, the zebrafish in the fasting group suffered higher mortality than those in the control group. Meanwhile, the activity of ALP and lysozyme improved with starvation. Lysozyme is an important component in the innate immune system of fish and takes part in the process of phagocytosis [50]. According to the results, starvation caused the mRNA expression of *il-1β*, *nfκb1*, *tgfb1a*, *tgfb1b* and *arg2*. These changes may be caused by oxidative stress. TGFB1 is a cytokine that has both pro- and anti-inflammatory effects [51]. Recombinant TGFB1 showed immunosuppressive activity and downregulated the expression of pro-inflammatory cytokines in leucocyte of teleosts. In zebrafish, the expression of *tgfb1a* caused inflammation in the liver [52]. Chronic tgfb1a induction activated inflammation, and its expression induced liver fibrosis in early hepatocellular carcinoma in zebrafish. Arginase is a primordial enzyme and is responsible for converting L-arginine into L-ornithine and urea. Arginase has two isoenzymes (arginase Ⅰ and arginase Ⅱ) [53,54]. Arginase Ⅱ is an immune-related gene and was found to be related to proinflammatory responses in macrophages via mitochondrial ROS. These data showed that starvation can induce an immune response in the kidney. In mice, starvation decreased the sensitivity to LPS and did not influence the expression of *il-1β* [55]. However, zebrafish stimulated with LPS exhibited a rough response to LPS, and the expression of *il-1β* and *mmp9* was significantly higher than in the other groups. In contrast to mice, zebrafish became more sensitive to LPS after starvation, so starvation may impair their ability to clear pathogens.

## 5. Conclusions

In the present study, we preliminarily explored the influence of fasting and refeeding on the innate immune system of zebrafish. Compensatory growth was realized in zebrafish, accompanied by increased susceptibility to pathogens after starvation. Oxidative stress—caused by starvation and the danger-associated molecular patterns produced by injured renal tubules—may have contributed to inflammation. Further study is needed to explore how starvation increases susceptibility and how the metabolic state influences different kinds of immune cells.

## Figures and Tables

**Table 1:** Feeding regime for each experiment.

Experiment	Feeding Regime
body weight	group1: feeding for 35 days; group2: starving for 7 days and refeeding for 28 days; group3: starving for 14 days and refeeding for 21 days; group4: starving for 21 days and refeeding for 14 days;
histopathology	F21: feeding for 21 days; S21: starving for 21 days; S21R3/5/7/14: starving for days and refeeding for 3/5/7/14 days;
biochemical parameter assay	F21/24/28: feeding for 21/24/28 days; S21/24/28: starving for 21/24/28 days; S21R3/7: starving for days and refeeding for3/7 days;
ROS production	
immune-related gene expression	
*Streptococcus agalactiae* challenge	F24: feeding for24 days; S24: starving for 24 days; S21R3/7: starving for days and refeeding for3/7 days;
LPS injection	
respiratory burst assay	

## Data Availability

Not applicable.

## Supplementary Materials

The following are available online at https://www.mdpi.com/article/10.3390/biom11060825/s1, Table S1: List of primers used in this study; Table S2: The statistic result of experiment.

## References

[1] Alwarawrah Y., Kiernan K., Maciver N.J. (2018). Changes in Nutritional Status Impact Immune Cell Metabolism and Function. Front. Immunol..

[2] Wu Z., Isik M., Moroz N., Steinbaugh M.J., Zhang P., Blackwell T.K. (2019). Dietary Restriction Extends Lifespan through Metabolic Regulation of Innate Immunity. Cell Metab..

[3] Wilson P.N., Osbourn D.F. (1960). Compensatory growth after undernutrition in mammals and birds. Biol. Rev..

[4] Ali M., Nicieza A., Wootton R.J. (2003). Compensatory growth in fishes: A response to growth depression. Fish. Fish..

[5] Bilton H.T., Robins G.L. (1973). The Effects of Starvation and Subsequent Feeding on Survival and Growth of Fulton Channel Sockeye Salmon Fry (*Oncorhynchus nerka*). J. Fish. Res. Board Can..

[6] Won E.T., Borski R.J. (2013). Endocrine regulation of compensatory growth in fish. Front. Endocrinol..

[7] Jobling M. (2009). Are compensatory growth and catch-up growth two sides of the same coin?. Aquac. Int..

[8] Whyte S.K. (2007). The innate immune response of finfish—A review of current knowledge. Fish. Shellfish. Immunol..

[9] Novoa B., Figueras A. (2012). Zebrafish: Model for the Study of Inflammation and the Innate Immune Response to Infectious Diseases. Curr. Top. Innate Immun..

[10] Mathis D., Shoelson S.E. (2011). Foreword Immunometabolism: An emerging frontier. Nat. Rev. Immunol..

[11] Walrand S., Moreau K., Caldefie F., Tridon A., Chassagne J., Portefaix G., Cynober L., Beaufrère B., Vasson M.-P., Boirie Y. (2001). Specific and nonspecific immune responses to fasting and refeeding differ in healthy young adult and elderly persons. Am. J. Clin. Nutr..

[12] Oarada M., Kamei K., Gonoi T., Tsuzuki T., Toyotome T., Hirasaka K., Nikawa T., Sato A., Kurita N. (2009). Beneficial effects of a low-protein diet on host resistance to Paracoccidioides brasiliensis in mice. Nutrition.

[13] Morris H.J., Carrillo O.V., Llauradó G., Alonso M.E., Bermúdez R.C., Lebeque Y., Fontaine R., Soria N.E., Venet G. (2010). Effect of starvation and refeeding on biochemical and immunological status of Balb/c mice: An experimental model of malnutrition. Immunopharmacol. Immunotoxicol..

[14] Petersen P.S., Lei X., Seldin M.M., Rodriguez S., Byerly M.S., Wolfe A., Whitlock S., Wong G.W. (2014). Dynamic and extensive metabolic state-dependent regulation of cytokine expression and circulating levels. Am. J. Physiol. Integr. Comp. Physiol..

[15] Li T., Qi M., Gatesoupe F.-J., Tian D., Jin W., Li J., Lin Q., Wu S., Li H. (2018). Adaptation to Fasting in Crucian Carp (*Carassius auratus*): Gut Microbiota and Its Correlative Relationship with Immune Function. Microb. Ecol..

[16] Martin S.A.M., Douglas A., Houlihan D.F., Secombes C.J. (2010). Starvation alters the liver transcriptome of the innate immune response in Atlantic salmon (*Salmo salar*). BMC Genom..

[17] Najafi A., Salati A.P., Yavari V., Asadi F. (2015). Effects of short term fasting and refeeding on some hematological and immune parameters in Mesopotamichthys sharpeyi (Gunther, 1874) fingerlings. Iran J. Sci. Technol. A.

[18] Gimbo R.Y., Favero G.C., Montoya L.N.F., Urbinati E.C. (2015). Energy deficit does not affect immune responses of experimentally infected pacu (*Piaractus mesopotamicus*). Fish. Shellfish. Immunol..

[19] Caruso G., Denaro M.G., Caruso R., Mancari F., Genovese L., Maricchiolo G. (2011). Response to short term starvation of growth, haematological, biochemical and non-specific immune parameters in European sea bass (*Dicentrarchus labrax*) and blackspot sea bream (*Pagellus bogaraveo*). Mar. Environ. Res..

[20] Biller J.D., Takahashi L.S. (2018). Oxidative stress and fish immune system: Phagocytosis and leukocyte respiratory burst activity. Anais Acad. Brasileira Ciências.

[21] Gill R., Tsung A., Billiar T.R. (2010). Linking oxidative stress to inflammation: Toll-like receptors. Free. Radic. Biol. Med..

[22] Li L., Chen Y., Gibson S.B. (2013). Starvation-induced autophagy is regulated by mitochondrial reactive oxygen species leading to AMPK activation. Cell. Signal..

[23] Wu C.-A., Chao Y., Shiah S.-G., Lin W.-W. (2013). Nutrient deprivation induces the Warburg effect through ROS/AMPK-dependent activation of pyruvate dehydrogenase kinase. Biochim. Biophys. Acta (BBA) Bioenerg..

[24] Renshaw S.A., Trede N.S. (2012). A model 450 million years in the making: Zebrafish and vertebrate immunity. Dis. Model. Mech..

[25] Vaidya V.S., Ozer J.S., Dieterle F., Collings F.B., Ramirez V., Troth S., Muniappa N., Thudium D., Gerhold D., Holder D.J. (2010). Kidney injury molecule-1 outperforms traditional biomarkers of kidney injury in preclinical biomarker qualification studies. Nat. Biotechnol..

[26] Yin W., Naini S.M., Chen G., Hentschel D.M., Humphreys B.D., Bonventre J.V. (2015). Mammalian Target of Rapamycin Mediates Kidney Injury Molecule 1-Dependent Tubule Injury in a Surrogate Model. J. Am. Soc. Nephrol..

[27] Craig P.M., Moon T.W. (2011). Fasted zebrafish mimic genetic and physiological responses in mammals: A model for obesity and diabetes?. Zebrafish.

[28] Jia J., Zhang Y., Yuan X., Qin J., Yang G., Yu X., Wang B., Sun C., Li W. (2018). Reactive oxygen species participate in liver function recovery during compensatory growth in zebrafish (Danio rerio). Biochem. Biophys. Res. Commun..

[29] Luo Z.-W., Jiang Y.-H., Lin L.-B., Deng X.-Y., Zhang Q. (2021). Genome-wide differential expression analysis explores antibacterial molecular mechanisms of zebrafish intestine upon pathogenic Streptococcus agalactiae challenge. Aquac. Rep..

[30] Li W., Su Y.-L., Mai Y.-Z., Li Y.-W., Mo Z.-Q., Li A.-X. (2014). Comparative proteome analysis of two Streptococcus agalactiae strains from cultured tilapia with different virulence. Vet. Microbiol..

[31] Goody M.F., Peterman E., Sullivan C., Kim C.H. (2013). Quantification of the respiratory burst response as an indicator of innate immune health in zebrafish. J. Vis. Exp..

[32] Hermann A.C., Millard P.J., Blake S.L., Kim C.H. (2004). Development of a respiratory burst assay using zebrafish kidneys and embryos. J. Immunol. Methods.

[33] Chomczynski P., Sacchi N. (1987). Single-step method of RNA isolation by acid guanidinium thiocyanate-phenol-chloroform extraction. Anal. Biochem..

[34] Livak K.J., Schmittgen T.D. (2001). Analysis of relative gene expression data using real-time quantitative PCR and the 2(-Delta Delta C(T)) Method. Methods.

[35] Bennett C.M., Kanki J.P., Rhodes J., Liu T.X., Paw B.H., Kieran M.W., Langenau D.M., Delahaye-Brown A., Zon L.I., Fleming M.D. (2001). Myelopoiesis in the zebrafish, Danio rerio. Blood.

[36] Traver D., Paw B.H., Poss K.D., Penberthy W.T., Lin S., Zon L.I. (2003). Transplantation and in vivo imaging of multilineage engraftment in zebrafish bloodless mutants. Nat. Immunol..

[37] Tang Q., Iyer S., Lobbardi R., Moore J.C., Chen H., Lareau C., Hebert C., Shaw M.L., Neftel C., Suva M.L. (2017). Dissecting hematopoietic and renal cell heterogeneity in adult zebrafish at single-cell resolution using RNA sequencing. J. Exp. Med..

[38] Agius C., Roberts R.J. (1981). Effects of starvation on the melano-macrophage centres of fish. J. Fish. Biol..

[39] Steinel N.C., Bolnick D.I. (2017). Melanomacrophage Centers as a Histological Indicator of Immune Function in Fish and Other Poikilotherms. Front. Immunol..

[40] Hamamura M., Oshikata T., Katoku K., Tsuchitani M., Yamaguchi R. (2017). Two types of deposits, hyaline droplets and eosinophilic bodies, associated with α2u-globulin accumulation in the rat kidney. J. Toxicol. Pathol..

[41] Decker J.H., Dochterman L.W., Niquette A.L., Brej M. (2012). Association of Renal Tubular Hyaline Droplets with Lymphoma in CD-1 Mice. Toxicol. Pathol..

[42] Cai J., Jiao X., Luo W., Chen J., Xu X., Fang Y., Ding X., Yu X. (2019). Kidney injury molecule-1 expression predicts structural damage and outcome in histological acute tubular injury. Ren. Fail..

[43] Sies H., Berndt C., Jones D.P. (2017). Oxidative Stress. Annu. Rev. Biochem..

[44] Reuter S., Gupta S.C., Chaturvedi M.M., Aggarwal B.B. (2010). Oxidative stress, inflammation, and cancer: How are they linked?. Free Radic. Biol. Med..

[45] Fukai T., Ushio-Fukai M. (2011). Superoxide Dismutases: Role in Redox Signaling, Vascular Function, and Diseases. Antioxid. Redox Signal..

[46] Tsikas D. (2017). Assessment of lipid peroxidation by measuring malondialdehyde (MDA) and relatives in biological samples: Analytical and biological challenges. Anal. Biochem..

[47] Morales A.E., Pérez-Jiménez A., Hidalgo M.C., Abellán E., Cardenete G. (2004). Oxidative stress and antioxidant defenses after prolonged starvation in Dentex dentex liver. Comp. Biochem. Physiol. Part. C.

[48] Hammock B.G., Ramírez-Duarte W.F., Garcia P.A.T., Schultz A.A., Avendano L.I., Hung T.-C., White J.R., Bong Y.-T., Teh S.J. (2020). The health and condition responses of Delta Smelt to fasting: A time series experiment. PLoS ONE.

[49] Liu X., Shi H., He Q., Lin F., Wang Q., Xiao S., Dai Y., Zhang Y., Yang H., Zhao H. (2020). Effect of starvation and refeeding on growth, gut microbiota and non-specific immunity in hybrid grouper (*Epinephelus fuscoguttatus* female x *E. lanceolatus* male). Fish Shellfish Immun..

[50] Saurabh S., Sahoo P.K. (2008). Lysozyme: An important defence molecule of fish innate immune system. Aquac. Res..

[51] Rebl A., Goldammer T. (2018). Under control: The innate immunity of fish from the inhibitors’ perspective. Fish. Shellfish. Immunol..

[52] Yan C., Yang Q., Gong Z. (2019). Transgenic expression of tgfb1a induces hepatic inflammation, fibrosis and metastasis in zebrafish. Biochem. Biophys. Res. Commun..

[53] Ming X., Rajapakse A.G., Yepuri G., Xiong Y., Carvas J., Ruffieux J., Scerri I., Wu Z., Popp K., Li J. (2012). Arginase II Promotes Macrophage Inflammatory Responses Through Mitochondrial Reactive Oxygen Species, Contributing to Insulin Resistance and Atherogenesis. J. Am. Heart Assoc..

[54] Song J., Eghan K., Lee S., Park J.-S., Yoon S., Pimtong W., Kim W.-K. (2020). A Phenotypic and Genotypic Evaluation of Developmental Toxicity of Polyhexamethylene Guanidine Phosphate Using Zebrafish Embryo/Larvae. Toxics.

[55] Faggioni R., Moser A., Feingold K.R., Grunfeld C. (2000). Reduced leptin levels in starvation increase susceptibility to endotoxic shock. Am. J. Pathol..

