# Supplementary material for: Innate Immune Response to Fasting and Refeeding in the Zebrafish Kidney
Source: Biomolecules. 2021 Jun 1;11(6):825. doi: 10.3390/biom11060825 (PMC8229452; doi:10.3390/biom11060825)
Supplement: Supplementary file 1 [file biomolecules-11-00825-s001.zip › biomolecules-1195869-supplementary.pdf]

Table S1 List of primers used in this study

qRT-PCR Primer	Primer sequence (5′ to 3′) (Forward)	Primer sequence (5′ to 3′) (Reverse)	ID
ef1α	GCTCGTTTTGAGGAAATCACC	CCATCCTGAAATTGGGACGAA	AY422992.1
nfκb1	TGAGTAAAGTGTTGGAGGAGC	GTCAGGCAGGAACGCAGT	NM_001353873
nfκb2	GATGAGAACGGAGACACGC	CTCTACTAACTTGGGTTGCTT	NM_001001840.3
arg2	CGGCGGACTGACCTACA	CCAGAGCGGATGCAACTA	NM_199611.3
havcr1	AGTTCTTGTGCCTGTTCTC	ACTGTTCGTATTCGCTTT	NM_001002434.1
il-1β	CTCGTACTCAAGGAGATC	CAGCTCGAAGTTAATGATG	NM_212844.2
tgfb1a	CCAGCAGAGCACGGATAAG	AGAGTTGCCATTTGAAGG	NM_182873.1
tgfb1b	AACTCACGCTTCATTTCCCTTC	CTTGGTCCGCTAAGAGGC	XM_687246.8
mmp9	CCTGCCAAATCAAGGAGT	ACCTTTGCGTTCACCATT	AY151254.1
nrf2a	GGAAGTCCATAACCGAAGAC	TGGGAATATCTCATTGTAATCTGAC	NM_182889

Table S2 The statistic result of experiment

Figure	group	Test of normality (normal distributions or not)	df	F-vlaue	Hmogeneity of varience test (variance equal or not)	parametric tests	post hoc multiple comparisons	p-value
Figure 1 A	7 days	Yes	42	2.221	Yes	t-test	-	p=0.002
	14 days	No	44	-	-	Mann-Whitney U	-	p=0.205
Figure 1 B	7 days	No	44	-	-	Mann-Whitney U	-	p=0.008
	14 days	No	44	-	-	Mann-Whitney U	-	p=0.000
	21 days	No	44	-	-	Mann-Whitney U	-	p=0.044
	28 days	No	44	-	-	Mann-Whitney U	-	p=0.380
Figure 1 C	7 days	No	44	-	-	Mann-Whitney U	-	p=0.015

	14 days	Yes	41	0.083	Yes	t-test	-	p=0.000
	21 days	Yes	41	1.705	Yes	t-test	-	p=0.000
	28 days	Yes	41	1.49	Yes	t-test	-	p=0.007
	35 days	Yes	41	1.49	Yes	t-test	-	p=0.457
Figure 1 D	-	Yes	8	3.331	Yes	one-way ANOVA	Bonferroni	F24 - S24: p=0.143<br>F24 - S21R3: p=0.337<br>S24 - S21R3: p=1.000
Figure 2 B	-	No	22	-	-	Kruskal-Wallis	-	S21R3 - S21R14: p=0.048<br>S21R5 - S2121R14: p=0.01
Figure 2 C	-	No	22		-	Kruskal-Wallis	-	p=0.054
Figure 2 D	F21 and S21	Yes	18	1.247	Yes	t-test	-	p=0.055
	F24, S24 and S21R3	Yes	28	5.343	No	Dunnett's T3	-	F24 - S24: p=0.064<br>F24 - S21R3: p=1.000<br>S24 - S21R3: p=0.070
	F28, S28 and S21R7	No	30	-	-	Kruskal-Wallis	-	F28 - S28: p=0.015<br>F28 - S21R7: p=1.000<br>S28 - S21R7: p=0.015
Figure 3 A	F21 and S21	Yes	13	0.086	Yes	t-test	-	p=0.046
	F24, S24 and S21R3	Yes	23	5.526	Yes	one-way ANOVA	Bonferroni	F24 - S24: p=0.057<br>F24 - S21R3: p=1.000<br>S24 - S21R3: p=0.016
	F28, S28 and S21R7	No	24	-	-	Kruskal-Wallis	-	F28 - S28: p=0.208<br>F28 - S21R7: p=0.103<br>S28 - S21R7: p=0.002
Figure 3 B	F21 and S21	No	20	-	-	Mann-Whitney U	-	p=0.019

	F24, S24 and S21R3	No	27	-	-	Kruskal-Wallis	-	F24 - S24: p=0.000<br>F24 - S21R3: p=0.041<br>S24 - S21R3: p=0.413
	F28, S28 and S21R7	No	25	-	-	Kruskal-Wallis	-	p=0.085
Figure 3 C	F21 and S21	No	18	-	-	Mann-Whitney U	-	p=0.040
	F24, S24 and S21R3	No	26	-	-	Mann-Whitney U	-	p=0.646
	F28, S28 and S21R7	Yes	27	0.824	Yes	one-way ANOVA	Bonferroni	p=450
Figure 4 A	F21 and S21	Yes	18	8.927	No	t-test	-	p=0.000757
	F24, S24 and S21R3	Yes	25	59.978	No	one-way ANOVA	Dunnet's T3	F24 - S24: p=0.000<br>F24 - S21R3: p=0.000<br>S24 - S21R3: p=0.000
	F28, S28 and S21R7	No	24	-	-	Kruskal-Wallis	-	F28 - S28: p=0.000<br>F28 - S21R7: p=0.402<br>S28 - S21R7: p=0.002
Figure 4 B	F21 and S21	Yes	13	0.797	Yes	t-test	-	p=0.102
	F24, S24 and S21R3	Yes	23	9.558	Yes	one-way ANOVA	Bonferroni	F24 - S24: p=0.006<br>F24 - S21R3: p=1.000<br>S24 - S21R3: p=0.002
	F28, S28 and S21R7	No	30	-	-	Kruskal-Wallis	-	F28 - S28: p=0.002<br>F28 - S21R7: p=0.780<br>S28 - S21R7: p=0.004
Figure 4 C	F21 and S21	Yes	18	2.646	Yes	t-test		p=0.004
	F24, S24 and S21R3	Yes	29	12.828	Yes	one-way ANOVA	Bonferroni	F24 - S24: p=0.000<br>F24 - S21R3: p=0.038<br>S24 - S21R3: p=0.072

	F28, S28 and S21R7	Yes	29	8.824	Yes	one-way ANOVA	Bonferroni	F28 - S28: p=0.001<br>F28 - S21R7: p=0.066<br>S28 - S21R7: p=0.273
Figure 5 A	F21 and S21	Yes	18	0.337	Yes	t-test	-	p=0.000
	F24, S24 and S21R3	No	26	-	-	Kruskal-Wallis	-	F24 - S24: p=0.003<br>F24 - S21R3: p=0.220<br>S24 - S21R3: p=0.537
	F28, S28 and S21R7	No	30	-	-	Kruskal-Wallis	-	F28 - S28: p=0.002<br>F28 - S21R7: p=0.310<br>S28 - S21R7: p=0.037
Figure 5 B	F21 and S21	Yes	13	2.746	Yes	t-test	-	p=0.029
	F24, S24 and S21R3	Yes	26	12.225	Yes	one-way ANOVA	Bonferroni	F24 - S24: p=0.000<br>F24 - S21R3: p=0.629<br>S24 - S21R3: p=0.006
	F28, S28 and S21R7	No	27	-	-	Kruskal-Wallis	-	F28 - S28: p=0.007<br>F28 - S21R7: p=0.447<br>S28 - S21R7: p=0.048
Figure 5 C3	F24, S24 and S21R3	No	36	-	-	Kruskal-Wallis	-	p=0.490
Figure 6 A	F21 and S21	No	16	-	-	Mann-Whitney U	-	p=0.000
	F24, S24 and S21R3	No	26	-	-	Kruskal-Wallis	-	F24 - S24: p=0.000<br>F24 - S21R3: p=0.083<br>S24 - S21R3: p=0.069
	F28, S28 and S21R7	No	30	-	-	Kruskal-Wallis	-	F28 - S28: p=0.000<br>F28 - S21R7: p=0.648<br>S28 - S21R7: p=0.002
Figure 6 B	F21 and S21	Yes	18	1.341	Yes	t-test	-	p=0.070

	F24, S24 and S21R3	Yes	28	8.845	Yes	one-way ANOVA	Bonferroni	F24 - S24: p=0.006<br>F24 - S21R3: p=1.000<br>S24 - S21R3: p=0.002
	F28, S28 and S21R7	No	30	-	-	Kruskal-Wallis	-	p=0.121
Figure 6 C	F21 and S21	Yes	16	3.917	Yes	t-test	-	p=0.084
	F24, S24 and S21R3	Yes	28	1.723	Yes	one-way ANOVA	Bonferroni	F24 - S24: p=0.252<br>F24 - S21R3: p=0.576<br>S24 - S21R3: p=1.000
	F28, S28 and S21R7	Yes	29	3.629	Yes	one-way ANOVA	Bonferroni	F28 - S28: p=0.065<br>F28 - S21R7: p=1.000<br>S28 - S21R7: p=0.106
Figure 6 D	F21 and S21	No	20	-	-	Mann-Whitney U	-	p=0.063
	F24, S24 and S21R3	No	29	-	-	Kruskal-Wallis	-	p=0.141
	F28, S28 and S21R7	No	30	-	-	Kruskal-Wallis	-	F28 - S28: p=0.002<br>F28 - S21R7: p=0.703<br>S28 - S21R7: p=0.006
Figure 6 E	F21 and S21	Yes	18	0.509	Yes	t-test	-	p=0.003
	F24, S24 and S21R3	No	30	-	-	Kruskal-Wallis	-	F24 - S24: p=0.002<br>F24 - S21R3: p=0.521<br>S24 - S21R3: p=0.010
	F28, S28 and S21R7	No	30	-	-	Kruskal-Wallis	-	F28 - S28: p=0.003<br>F28 - S21R7: p=0.461<br>S28 - S21R7: p=0.024
Figure 6 F	F21 and S21	Yes	18	1.964	Yes	t-test	-	p=0.001

	F24, S24 and S21R3	No	30	-	-	Kruskal-Wallis	-	F24 - S24: p=0.038<br>F24 - S21R3: p=0.046<br>S24 - S21R3: p=0.000
	F28, S28 and S21R7	No	30	-	-	Kruskal-Wallis	-	F28 - S28: p=0.007<br>F28 - S21R7: p=0.477<br>S28 - S21R7: p=0.045
Figure 7 A	F24, PBS-LPS	Yes	20	13.698	No	t-test	-	p=0.000
	S24, PBS-LPS	No	18	-	-	Mann-Whitney U	-	p=0.006
	R24, PBS-LPS	Yes	22	6.391	No	t-test	-	p=0.0001
	PBS	No	30	-	-	Kruskal-Wallis	-	F24 - S24: p=0.007<br>F24 - S21R3: p=0.025<br>S24 - S21R3: p=0.564
	LPS	No	32	-	-	Kruskal-Wallis	-	p=0.089
Figure 7 B	F24, PBS-LPS	Yes	20	0.528	Yes	t-test	-	p=0.009
	S24, PBS-LPS	Yes	16	2.551	Yes	t-test	-	p=0.248
	R24, PBS-LPS	No	18	-	-	Mann-Whitney U	-	p=0.004
	PBS	Yes	31	6.225	Yes	one-way ANOVA	Bonferroni	F24 - S24: p=0.159<br>F24 - S21R3: p=0.503<br>S24 - S21R3: p=0.004
	LPS	Yes	31	0.673	Yes	one-way ANOVA	Bonferroni	F24 - S24: p=1.000<br>F24 - S21R3: p=1.000<br>S24 - S21R3: p=0.827
Figure 7 C	F24, PBS-LPS	Yes	20	0.396	Yes	t-test	-	p=0.718
	S24, PBS-LPS	No	18	-	-	Mann-Whitney U	-	p=0.055
	R24, PBS-LPS	No	18	-	-	Mann-Whitney U	-	p=1.000
	PBS	No	32	-	-	Kruskal-Wallis	-	p=0.055

	LPS	No	32	-	-	Kruskal-Wallis	-	F24 - S24: p=0.235<br>F24 - S21R3: p=0.041<br>S24 - S21R3: p=0.003
